# Multilayer Stock Forecasting Model Using Fuzzy Time Series

**DOI:** 10.1155/2014/610594

**Published:** 2014-01-29

**Authors:** Hossein Javedani Sadaei, Muhammad Hisyam Lee

**Affiliations:** Department of Mathematical Sciences, Faculty of Science, Universiti Teknologi Malaysia, 81310 Skudai, Johor, Malaysia

## Abstract

After reviewing the vast body of literature on using FTS in stock market forecasting, certain deficiencies are distinguished in the hybridization of findings. In addition, the lack of constructive systematic framework, which can be helpful to indicate direction of growth in entire FTS forecasting systems, is outstanding. In this study, we propose a multilayer model for stock market forecasting including five logical significant layers. Every single layer has its detailed concern to assist forecast development by reconciling certain problems exclusively. To verify the model, a set of huge data containing Taiwan Stock Index (TAIEX), National Association of Securities Dealers Automated Quotations (NASDAQ), Dow Jones Industrial Average (DJI), and S&P 500 have been chosen as experimental datasets. The results indicate that the proposed methodology has the potential to be accepted as a framework for model development in stock market forecasts using FTS.

## 1. Introduction

The statistical complex system model investigation of financial market index and return is an issue to understand and model the distribution of financial price fluctuation, which has long been an effort of economic study. As the stock markets are becoming deregulated globally, the stock market system modeling and forecast are becoming more complex in the risk management and derivatives rating. The development of novel statistical analyzing methods of stock returns delivers numerous observed indications of old random-walk hypothesis, demanding the invention of new financial models to describe price movements in the market [1]. However, one of the key aspects of complex statistical model in stock market is accurate forecasting that could yield significant profits and it could also decrease investment risks [2–4]. Considering the stock prediction, the most frequently used forecasting methods are nonlinear models, for example, neural network [5–7], genetic algorithm [8, 9], hybrid models [10–12], fuzzy logic [13], and support vector machine [14]. However, fuzzy time-series method has been developed as one of novel forecasting methods in this area. So far, various FTS have been applied successfully to handle stock index forecasting [15–23]. Since this study is focused on applying FTS on stock data prediction, the following paragraphs provide a brief review of FTS models.

Song and Chissom [24, 25] first applied a FTS model by using fuzzy relation equations and approximate reasoning. There are two classes of FTS: time-variant and time-invariant. Chen [26] presented a method to forecast student enrolment at the University of Alabama that takes less time computing max-min composition operations than Song and Chissom's model [24, 25].

The length of intervals influences forecast accuracy in FTS. Consequently, determining optimal length of interval in FTS is the central issue in studies. Along these lines, Huarng [27] proposed distribution- and average-based length to determine the effective length of intervals in FTS. In addition, Sheng and Yeh [28], at their work, presented a novel approach to handle the issue of finding the effective length by applying the natural partitioning technique, which can recursively partition the universe of discourse level by level in a natural way. They indicated that the model could be used to handle high-order FTS as well. Experimental results on the enrolment data of the University of Alabama proved that the results of forecasting model could forecast the data effectively and efficiently. Yu [29] proposed a refined fuzzy time-series model to further refine the lengths of intervals. Their model could improve the lengths of intervals during the formulation of fuzzy relationships and hence established the fuzzy relationships more appropriately. Using genetic algorithms, Chen and Chung [30] presented a method that modified the length of each interval in the universe of discourse to deal with the forecasting complications based on high-order fuzzy time. Moreover, they used historical enrolments of the University of Alabama to illustrate the forecasting process of their proposed method. Cheng et al. [16, 31] proposed two approaches for overcoming the problems of determining the universe of discourse, the length of intervals, and membership functions of FTS. Huarng and Yu [32] proposed ratio-based lengths of intervals to improve FTS forecasting. In their research, algebraic growth data, such as enrolments and the stock index, and exponential growth data, such as inventory demand, were selected as the forecasting targets. The empirical examination recommended that the ratio-based lengths of intervals could also be used to improve FTS forecasting. Li and Cheng [33] proposed a deterministic forecasting model to accomplish the issues of controlling uncertainty in forecasting, partitioning the intervals effectively, and consistently achieving forecasting accuracy with different interval lengths. In addition, in their work, an important parameter, the maximum length of subsequence in a FTS resulting in a certain state, was deterministically quantified. Moreover, their model followed the consistency principle that a shorter interval length leads to more accurate results. Later, Li et al. [34] proposed a novel forecasting model to enhance forecasting functionality and to allow processing of two-factor forecasting problems. Moreover, that model applied fuzzy c-means (FCM) clustering to deal with interval partitioning, which considered the nature of data and formed unequal-sized intervals. In a recent study, Wang and Chen [35] presented a method to forecast the temperature and the Taiwan Futures Exchange (TAIFEX) based on automatic clustering techniques and a two-factor, high-order FTS. Aladag et al. [36] proposed another approach which used a single-variable constrained optimization to determine the ratio for the length of intervals. Their method was successfully applied to the two case studies, which are the enrolment data at the University of Alabama and the inventory demand data. Su et al. proposed a model for the forecasting process, which combined two granulating methods (the minimize entropy principle approach and the cumulative probability distribution approach) and a rough set algorithm [37]. Their model surpassed the conventional fuzzy time-series models and a multiple regression model (MLR) in forecast accuracy. In a different study, Egrioglu et al. [38] proposed a new method which used MATLAB function that employs an algorithm based on golden section search to optimize a function with single-variable constraint for finding the effective length of intervals in high-order FTS. Forecasting number of enrolments in Alabama University showed a great improvement in accuracy by this method.

A remarkable development in forecasting stock market has been materialized by using adaption models. Few studies dealt with this issue. Cheng et al. [39], for instance, introduced a fuzzy time-series model which combines the adaptive expectation model into forecasting processes to adjust forecasting errors. Liu et al. [20] presented a multiple attribute FTS method, which integrates a clustering method and adaptive expectation model. Teoh et al. [21] proposed a hybrid model based on multiorder FTS, which employs rough sets theory to mine fuzzy logical relationship from time-series and adaptive expectation model to modify forecasting results in order to increase forecasting accuracy. Chen et al. [15] proposed a model that could adjust the forecasting results with the possibility of minimal error in the training dataset.

Based on the above information, most of forecasting literatures to date have focused on the development of specific algorithms. In addition, in some of these studies, the effect of data preprocessing has been disregarded; that is, researchers directly utilized unprocessed stock data for forecasting purposes. This gives the impression that they are not willing to spend time doing data preprocessing. However, as it will be noted in the following sections, forecast accuracy will be improved by appropriate data preprocessing.

The second issue is the shortage of forecasts modification based on recent observations. As stated above, adaption of forecast has major impacts on forecast accuracy; nonetheless, it has received consideration in few studies.

Still another vague issue in previous studies is determining universe of discourse and establishing linguistic variables. In order to forecast stock market using FTS, we need to determine the length of each interval to establish linguistic variables. Even though researchers proposed many approaches to reconcile this problem, almost all of them ignored to show how universe of discourse must be exactly defined. Besides distinguishing the length of intervals, another issue that must be taken into account is determining the starting point of universe of discourse. If the role of starting points is neglected in FTS algorithms, it will be difficult to judge whether a particular FTS model exactly produces robust forecast or not. To address the importance of the starting point as an important gap in previous studies, we perform certain experiments. The results demonstrate that while the lengths of intervals are identical, the degree of accuracy is considerably different with different starting points (notice Table 1). For instance, notice the difference in years 1991, 1992, and 1998. In this table, the starting point corresponding to each year is start = min⁡(data) − *D*1.

Last but not least, there is lack of studies on the combination of different algorithms having positive role in stock market prediction. In other words, the hybridization of constructive findings of earlier studies appears to receive less attention. Based on their interest, researchers focus on certain subjects such as developing FTS algorithms or developing algorithms for finding the effective length of intervals; however, there is not any systematic model to motivate them to combine or ensemble several positive features to progress forecasts in this field of study.

In this study, our approach differs from those reviewed in the literature. Earlier studies were tied to a particular algorithm. However, in this study, the aim is not to propose new algorithm; instead, we propose a systematic, descriptive, and well-structured framework model, which is constructed of some meaningful layers that play an independent role throughout the forecast process. Each layer has a responsibility to resolve specific problems such as those mentioned above. The proposed methodology is model-based rather than algorithm-based.

The rest of the paper proceeds as follows. The next section presents related works to FTS models. In Section 3, the framework of proposed multilayer stock forecasting model is documented.  Section 4 provides information about stock databases that used in this study. In Section 5 empirical experiments are presented.  Section 6 provides our discussions and findings. The final section displays conclusions and future works.

## 2. Related Studies

This section provides definitions of fuzzy time series. Furthermore, weighted fuzzy time-series algorithm is explained.

### 2.1. Fuzzy Time-Series Definitions and Algorithms

Song and Chissom first presented the concepts of fuzzy time series [24, 25, 41], where the values in a fuzzy time series are presented by fuzzy sets [42]. Let *U* be the universe of discourse, where *U* = {*u*_1_, *u*_2_,…, *u*_*m*_}. A fuzzy set *A*_*i*_  (*i* = 1,2,…, *m*) of *U* is defined as follows:

(1) Ai=fAi(u1)u1+fAi(u2)u2+⋯+fAi(um)um,

where *f*_*A*_*i*__ is the membership function of the fuzzy set *A*_*i*_, *f*_*A*_*i*__ : *U* → [0,1]; *u*_*n*_ is a generic element of fuzzy set *A*_*i*_; *f*_*A*_*i*__(*u*_*n*_) is the degree of belongingness of *u*_*n*_ to *A*_*i*_; *f*_*A*_*i*__(*u*_*n*_)∈[0,1] and 1 ≤ *n* ≤ *m*.

**Definition 1 (see [25])**

Let *Y*(*t*), *t* = …, 0,1, 2,…, a subset of real numbers *R*, be the universe of discourse by which fuzzy sets*f*_*j*_(*t*) are defined. If *F*(*t*) is a collection of *f*_1_(*t*), *f*_2_(*t*),…, then *F*(*t*) is called a fuzzy time series defined on *Y*(*t*).

**Definition 2 (see [25])**

If there exists a fuzzy relationship *R*(*t* − 1, *t*), such that *F*(*t*) = *F*(*t* − 1)∘*R*(*t* − 1, *t*), where “∘” is an arithmetic operator, then *F*(*t*) is said to be caused by *F*(*t* − 1). The relationship between *F*(*t*) and *F*(*t* − 1) can be denoted by *F*(*t* − 1) → *F*(*t*).

**Definition 3 (see [25])**

Suppose that *F*(*t*) is calculated only by *F*(*t* − 1) and *F*(*t*) = *F*(*t* − 1)∘*R*(*t* − 1, *t*). For any *t*, if *R*(*t* − 1, *t*) is independent of *t*, then *F*(*t*) is considered a time-invariant fuzzy time series. Otherwise, *F*(*t*) is time-variant. Assuming *F*(*t* − 1) = *A*_*i*_ and *F*(*t*) = *A*_*j*_, a fuzzy logical relationship can be defined as *A*_*i*_ → *A*_*j*_, where *A*_*i*_ and *A*_*j*_ are called the left-hand side (LHS) and right-hand side (RHS) of the fuzzy logical relationship, respectively.

### 2.2. The Algorithm of Yu's Model

Since in this study we employ weighted FTS proposed by Yu [22], in this section we stated it with details as follows.

**Step 1**

Defining the universe of discourse and intervals for observations: according to the problem domain, the universe of discourse for observations is defined as *U* = [starting, ending]. The length of the intervals, *l*, is then determined, where *U* can be partitioned into equal-length intervals *u*_1_, *u*_2_,…, *u*_*b*_. Each interval *u*_*d*_ can be considered to be [starting + (*d* − 1) × *l*, starting + *d* × *l*] and each matching midpoint *M*_*d*_ as 1/2 × (starting + (*d* − 1) × *l* + starting + *d* × *l*) where *d* = 1,2, 3,…, *m*.

**Step 2**

Defining fuzzy sets for observations: each linguistic observation *A*_*i*_ can be defined by the intervals *u*_1_, *u*_2_, …, *u*_*m*_.   *A*_*i*_ = *f*_*Ai*_(*u*_1_)/*u*_1_ + *f*_*Ai*_(*u*_2_)/*u*_2_ + ⋯+*f*_*Ai*_(*u*_*m*_)/*u*_*m*_. Each *A*_*i*_ can be denoted as *A*_*i*_ = ⋯+0/*u*_*i*−2_ + 0.5/*u*_*i*−1_ + 1/*u*_*i*_ + 0.5/*u*_*i*+1_ + 0/*u*_*i*+2_ + ⋯, *i* = 1,2,…, *m*.

**Step 3**

Fuzzifying each observations in training dataset.

**Step 4**

Establishing FLRs: two successive fuzzy sets, *A*_*i*_ (at *t* − 1) and *A*_*j*1_ (at *t*), can be used to create the FLR *A*_*i*_ → *A*_*j*1_.

**Step 5**

Establishing fuzzy relationships: the FLRs with similar LHS establish FLRGs.

**Step 6**

Forecasting: supposing the FLRG *A*_*i*_ → *A*_*j*1_, *A*_*j*2_,…, *A*_*jk*_. (1 ≤ *k* ≤ *m*), if *F*(*t* − 1) = *A*_*i*_, then the forecast of *F*(*t*) is *A*_*j*1_, *A*_*j*2_,…, *A*_*jk*_.

**Step 7**

Defuzzifying: assume that the forecast of *F*(*t*) is *A*_*j*1_, *A*_*j*2_,…, *A*_*jk*_. The defuzzified matrix is equivalent to a matrix with midpoints *M*(*t*) = [*M*_*j*1_, *M*_*j*2_,…, *M*_*jk*_], where *M*(*t*) denotes the defuzzified forecast of *F*(*t*).

**Step 8**

Assigning weights: assume the forecast of *F*(*t*) is *A*_*j*1_, *A*_*j*2_,…, *A*_*jk*_. The corresponding weights for *A*_*j*1_, *A*_*j*2_,…, *A*_*jk*_, for example, *w*_1_, *w*_2_,…, *w*_*k*_, are stated. Before making the weight matrix with these *w*_1_, *w*_2_,…, *w*_*k*_, the weight matrix *W*(*t*) = [*w*_1_′, *w*_2_′,…, *w*_*k*_′] must satisfy the condition ∑_*h*=1_^*k*^ *w*_*h*_′ = 1. Henceforth, these weights *w*_1_, *w*_2_,…, *w*_*k*_ should be standardized. Then achieve the next weight matrix:

(2) W(t)=[w1′,w2′,…,wk′]=[w1∑h=1kwh,w2∑h=1kwh,…,wk∑h=1kwh],

where *w*_*h*_ is the corresponding weight for *A*_*jh*_. Furthermore, the weight matrix is monotonic; therefore, it also satisfies the following condition:

(3) $$\begin{array}{c} w_{1}\leq w_{2}\leq \cdots \leq w_{k}. \end{array}$$

One intuitive weight scheme based on Yu's study is here:

(4) $$\begin{array}{c} w_{1}=1,\;\;w_{i}=w_{i-1}+1\;\text{for}\;\;i\geq 2. \end{array}$$

Hence, the *i*th item in (2) can be denoted as

(5) W(t)=[w1′,w2′,…,wk′]=[1∑h=1kh,2∑h=1kh,…,h∑h=1kh].

**Step 9**

Calculating results: in the weighted model, the final forecast is equal to the product of the defuzzified matrix and the transpose of the weight matrix: final(*t*) = *M*(*t*) × *W*(*t*)^*T*^, where “×” is the matrix product operator, *M*(*t*) is a 1 × *k* matrix, and *W*(*t*)^*T*^ is a *k* × 1 matrix.

## 3. The Framework of the Proposed Multilayer Stock Forecasting Model 

**Figure 1:**
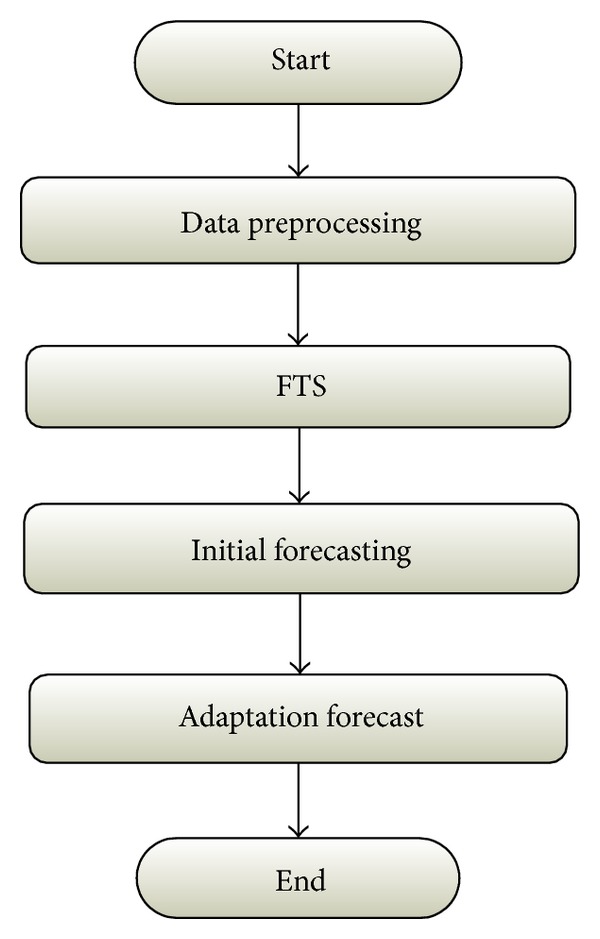
Proposed multilayer model.

Having discussed the key points in the introduction section, we proposed a multilayer model that could be beneficial for stock market forecasting by using FTS methods. The proposed model contains five logical meaningful layers as displayed in Figure 1.

Each layer has its specific task to assist forecast process by reconciling certain problems. The details about these layers are as follows.

*Layer 1*. Data preprocessing layer: in this layer, the aim is to transform original data to new domain with less fluctuation or volatility. For instance, detrendization of data assists in the forecastability increase [43, 44]. This layer is supposed to stabilize variance and mean of data that have major impact on forecasting. Likewise, detecting and handling outliers, filtering inconsistent data, and reducing noises are performed in this layer.

*Layer 2*. Universe of discourse and portioning: in this layer, the universe of discourse should be recognized. In addition, the number of linguistic variables and the number of intervals or the length of each interval used in FTS must be exactly determined. There are some advanced research works in this area of study, for example, [27, 32, 37, 38, 45, 46], and so forth. Previous studies imply the fact that development in this layer functionally has positive influence on forecast.

*Layer 3*. FTS: this layer is about deciding on the proper FTS method for stock data prediction. So far, different FTS algorithms have been adopted to forecast stock data, for example, [15–23, 37] and so forth. The more appropriate the FTS method is selected or developed, the more enhanced the whole model will be.

*Layer 4*. Initial forecasting: in this layer, initial forecast is calculated. The possibility to improve initial forecasts in this layer occurs by either using novel defuzzification methods [45], or employing additional information inside training datasets [23], or applying expertise knowledge [47], or giving appropriate weights in forecast process [22], and so forth.

*Layer 5*. Adaptation: in stock markets, investors usually make their investment decisions based on recent stock evidence, for example, late market news, stock technical indicators, or price fluctuations. Thus, it is logical that investors will modify their forecasts with the latest prediction errors [39]. The aim of this layer is using recent periods of forecasting errors to modify the forecast for the future stock index by employing adaptive expectation models.

In theory, the developments in all forecast procedure in the whole model will be promised by concentrating on and then advancing each layer. The ideal circumstance happens when every single layer could be categorically standing by itself. In other words, development in each layer can influence the improvement in forecast accuracy. In practice, interdependencies cannot be removed from among layers completely, since layers interact with each other. Nonetheless, the proposed multilayer model attempts to implement a perfect model as much as possible. Considering this hypothesis and based on researchers' interest, they are able to focus on improvement performance of layers exclusively. For instance, imagine that layers *A*, *B*, *C*, *D*, and *E* were already proposed, corresponding to five layers of perfect model, respectively. Suppose that new FTS algorithm is developed as *C*′ corresponding to FTS layer; thus, in theory, applying new sequence of layers, that is *A*, *B*, *C*′, *D*, and *E* together, can lead to further improvement in forecasting compared with the previous one. By this hypothesis, we perform huge experiments to check reliability and predictability strength of the proposed model.

## 4. Data 

To illustrate the proposed method, 10 years of closing prices data of TAIEX (Taiwan Stock Exchange Capitalization Weighted Stock Index), NASDAQ (National Association of Securities Dealers Automated Quotations) from 1990 to 1999, DJI (Dow Jones Industrial Average), and S&P 500 from 2000 to 2009 were chosen as experimental datasets. The first ten months (January–October) of each year were considered as training datasets and the remaining last months (November and December) as testing datasets.

## 5. Empirical Works 

As noted above, one of the key assumptions for model development is improving a particular layer separately. In this section, we use our experience, knowledge, and previous findings to propose suggestions to positively improve the layers gradually. The experiment process involves certain stages as follows.

(*1) Data Preprocessing.* As stated in Section 4, there are several motives to perform data preprocessing. This part used Return on Investment, ROI, concept for data preprocessing. It is the efficiency of an investment and use to compare the efficiency of a number of different investments:

(6) $$\begin{array}{c} \text{ROI}=\frac{\text{Gains}-\text{Investments}\;\;\text{Costs}}{\text{Investments}\;\;\text{Costs}}. \end{array}$$

Consider a time series such as {*X*_*t*_} which denotes a stock price of particular item *I*; then, we define daily ROI for this item as follows:

(7) $$\begin{array}{c} {\text{ROI}}_{t}=\frac{x_{t}\left(I\right)-x_{t-1}\left(I\right)}{x_{t-1}\left(I\right)}. \end{array}$$

Equation (7) provides strong criteria for investors to decide whether investment on item *I* is gainful or not. In all stock markets, the condition is similar; for example, TAIEX, NASDAQ, DJI, and S&P 500 indexes can be considered as reflection of overall market movement, because these indexes present the movement average of many individual stocks such as item *I*. By this brief introduction, we start our proposed data preprocessing. Assume {*Z*_*t*_} is time series of our interest in stock databases, namely, TAIEX, NASDAQ, DJI, or S&P 500; then, we define

(8) $$\begin{array}{c} \text{ROI}\left(t\right)=\frac{z\left(t\right)-z\left(t-1\right)}{z\left(t-1\right)}. \end{array}$$

**Figure 2:**
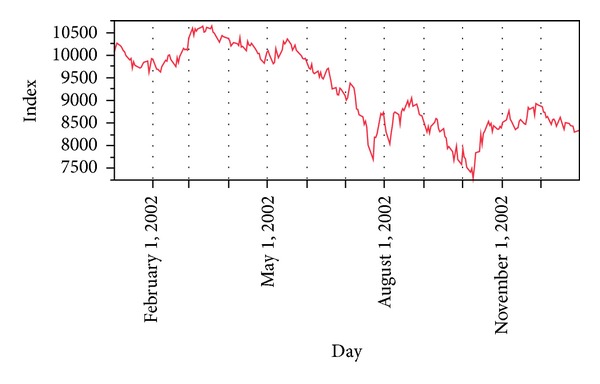
Unprocessed data for year 2002 of DJI.

**Figure 3:**
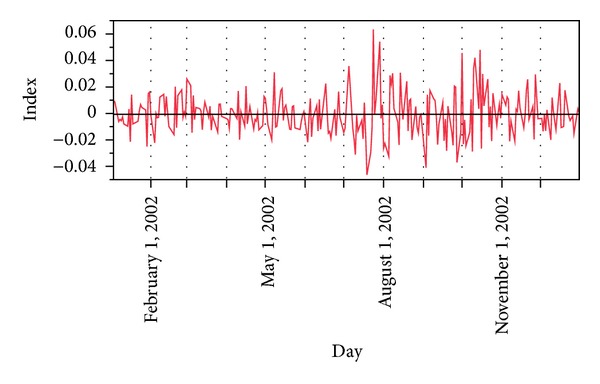
Processed data for year 2002 of DJI.

Hence, our proposed data preprocessing gives us new time series, that is, {ROI(*t*)} in new domain with less volatility and noisy effects. Notice and compare Figures 2 and 3.

Thus, for one-step-ahead forecasting, for example, at time *t* + 1, we employ and compare both {*Z*_*t*_} and {ROI(*t*)} to emphasis positive influence of proposed data preprocessing on forecasting.

(*2) Universe of Discourse and Partitioning.* to demonstrate improvement in forecast process stepwise, we initially employ the effective length of intervals based on Huarng's [27] findings. This study utilizes average-based length (notice Tables 2 and 3). These lengths are denoted by *θ* in all tables, and then to refine the accuracy further by minimizing error in fitting values, we try to find optimum length in the range [*θ* − 0.2*θ*, *θ* + 0.2*θ*] for each year. Next, we use optimum length for forecasts. The optimum lengths are displayed by θ*s. After performing data preprocessing based on (8), we use Sturges's [48] formula for calculating the effective lengths (see Tables 2 and 3), which is simply defined by

(9) $$\begin{array}{c} l=\frac{\max\left(\left\{\text{ROI}\left(t\right)\right\}\right)-\min\left(\text{ROI}\left(t\right)\right)}{\text{lo}{\text{g}}_{2}^{n}+1}, \end{array}$$

where *n* is the number of ROI(*t*) members.

Finally, like the above process, we find optimum length in the [*l* − 0.2*l*, *l* + 0.2*l*], which are denoted by l*s, and use them to improve forecasts.

(*3) FTS.* for model illustration, due to space limitations, we just choose Yu's (2005) FTS first-order algorithm which is frequently used in stock market forecasting area of researches [15, 21, 22, 49].

(*4) Initial Forecasting.* in our experiments, if data preprocessing is performed, then initial forecast retrieved by converting operation will be as follows:

(10) $$\begin{array}{c} \hat{z}\left(t+1\right)=z\left(t\right)\left(\text{R}\hat{\text{O}}\text{I}\left(t+1\right)+1\right). \end{array}$$

Otherwise, initial forecast is calculated as usual.

In the above equation, $\hat{z}(t+1)$ is initial forecast at time *t* + 1 and $\text{R}\hat{\text{O}}\text{I}(t+1)$ is forecasted value of ROI at time *t* + 1.

(*5) Adaption.* in the following experiments, two types of forecast modification are utilized. These equations are employed to adapt the initial forecast to promote better forecasts. Type I adaption is adopted from Cheng's [39] study which is defined as

(11) $$\begin{array}{c} \text{Adaptive}\_\text{forecast}\left(t+1\right)=p\left(t\right)+\alpha \left(\hat{z}\left(t+1\right)-p\left(t\right)\right) \end{array}$$

and type II is retrieved from Chen et al.'s [15] study which is derived by

(12) Adaptive_forecast(t+1)=p(t)+α(z^(t+1)−p(t))+β(z^(t)−p(t−1)),

where −1 ≤ *α*, *β* ≤ 1, $\hat{z}(t)$ is forecasted value and *p*(*t*) is actual value at time*t*.

### 5.1. Illustrative Experiments

To examine how the proposed model causes improvement in forecast procedure stepwise and to show to what extent the existence of each layer is meaningful, we design certain experiments in chronological order. However, the main idea in these experiments is to restrict attention to the role of three layers, namely, data preprocessing, universe of discourse, and adaption, in forecasts improvement functionally in the proposed multilayer model. In these experiments, the influence of the rest of layers on developing model is being of secondary concern.

In empirical works, first, we used unprocessed data without using adaption and then we employed both processed data and adaption layers. To simplify demonstration, all experiments are categorized in groups a and b as follows:

- databases: TAIEX, NASDAQ, DJI, and S&P 500;'
   data preprocessing: Ne;
   universe of discourse: starting = min⁡(data) − *D*1 (for TAIEX and DJI, D1 = 10 and for NASDAQ and S&P 500, D1 = 2);
   length of interval: both *θ*s and θ*s are employed to compare.
   adaption: Ne, type I, and type II;
   results are collected in Table 4;

(b) databases: TAIEX, NASDAQ, DJI, and S&P 500:
   data preprocessing: according to (8);
   universe of discourse: starting = min⁡(data) − 0;
   length of interval: both *l*s and l*s are employed to compare;
   adaption: Ne, type I and type II;
   results are collected in Table 5.

## 6.   Remarks, Findings, and Discussions of the Proposed Model

The root mean square error (RMSE) is a frequently used criterion in comparison results, which we also utilized in our proposed model. This criterion is defined as follows:

(13) $$\begin{array}{c} \text{RMSE}=\sqrt{\frac{\sum_{i=1}^{n}{\left[\text{forecasted}\_\text{value}\left(i\right)-\text{real}\_\text{value}\left(i\right)\right]}^{2}}{n}}. \end{array}$$

In this section, six key results of this empirical study are presented as follows.

1. The proposed model provides changes in stock market forecasting using FTS from the algorithm-oriented to the model-oriented, which is a further advanced view.
2. The results presented here show that the proposed data preprocessing together with the proposed effective length of intervals, that is, *l*s, has major positive effects on forecast accuracy in comparison with using unprocessed (original) data together with average-based length, that is, *θ*s. Consequently, as expected, presence of this layer in the proposed model was logical. For instance, while the average of RMSEs for 10 years TAIEX was 119.7 (Table 4, first row) by using unprocessed data, the average of RMSEs was interestingly dropped by 26.3 from 119.7 to 93.4 (Table 5, first row) by applying processed data and the effective length. The rest of the dropping amounts for all cases were 15.6, 15.7, 25.1, 16.4, 16.5, 1.6, 0.7, 0.7, 1.8, 1, 1.2, 47, 21.6, 21.3, 47, 25.3, 24.8, 2.6, 1.1, 1.2, 3.5, 2.1, and 2.2. In whole, in 80% of all experiments, the proposed data preprocessing together with the proposed length showed better forecasts.
3. Between two adaption types used in this research (typs I and II), type I provided superior RMSE performance. In 50% of all experiments, type I promote better forecast than type II. In 28% of all experiments, two methods produced similar results and only in 22% of all experiments, type II presented superior performance.
4. While applying preprocessed data, the results from these experiments, which are presented in Tables 4 and 5, reveal that employing Sturgis's formula for calculating effective length of intervals improves forecast accuracy.
5. Reviewing RMSEs in Tables 4 and 5 emphasize that using adaption layer in the proposed multilayer model has positive influence on forecast accuracy; therefore, the presence of this layer in the proposed model was reasonable.
6. Employing optimal length (in our study θ*s and l*s) slightly reduce RMSEs. Just in 56% of related experiments, using optimal lengths contributed to improve forecasts. Since finding these values is time-consuming, it is up to users whether they approve to pay extra costs to obtain them or to use *θ*s or *l*s directly.

## 7. Conclusions and Future Works

In this study, a five-layer model was proposed for stock market forecasting. The model was established based on this assumption that thinking of, improving, and advancing each layer separately will guarantee the development in the whole model. To check whether the proposed model is reliable and can promote enhancement in stock market forecasting, we designed experiments and performed enhancing in each layer gradually; however, the goal was to highlight the role of data preprocessing, the proposed effective length of intervals, and adaption layer. After comparing 480 different results, it was proved that the multilayer model is proper for model development and can therefore be used for stock market forecasting purposes. In short, although presenting a new model is not a definite proposition because not everyone will agree on the principles followed, the results show that the proposed model can be considered as a standard systematic model whereby it is possible to develop stock predictions by using FTS.

For future research, considering the behavior of each layer discretely as much as possible will lead to the development of layers more rapidly, because these roles might be captured by some specifications of the externally observable subsystems. In this way, many questions remain to be answered and many other problems remain to be researched to develop an improved version of the proposed model. Some critical questions for further studies, for instance, are in which order the development of layers should be carried out, which layers have more contribution to enhance the whole model, how it is possible to add more significant component to this model, and where should research efforts be directed to develop this model. In short, based on proficiencies and interests, researcher can develop performance of specific layer.

## Figures and Tables

**Table 1:** Forecasting performance for TAIEX with length = 100.

Model	*D*1	Year									
		1990	1991	1992	1993	1994	1995	1996	1997	1998	1999
Chen [40]	50	215.4	109.6	58.8	120.9	107.5	78.6	63.9	145.3	139.3	122.3
	70	217.9	96.9	67.7	125.6	106.7	92.9	57.7	148.4	125.7	123.2

Yu [17, 32]	50	219.7	77.3	52.3	113.6	102.9	74.2	65.1	140.6	145.7	115.8
	70	223.7	92.1	71.5	124.1	99.5	87.4	59.2	138.8	124.5	112.4

**Table 2:** Different length of intervals for TAIEX and NASDAQ problems.

Year	TAIEX				NASDAQ			
	*θ*	θ*	*l*	l*	*θ*	θ*	*l*	l*
1990	100	82	0.015111473	0.016389178	2	1.73	0.012644574	0.011515659
1991	70	59	0.023985040	0.019988032	2	1.66	0.009269332	0.007415466
1992	30	24	0.011033589	0.009826871	2	1.84	0.006353103	0.005482482
1993	30	25	0.014197516	0.011658013	2	1.68	0.006699332	0.006659466
1994	40	32	0.013543730	0.011834984	2	1.64	0.007258739	0.008306991
1995	30	25	0.011603234	0.009282587	3	2.60	0.007482858	0.006186286
1996	30	27	0.012010076	0.009608061	4	3.28	0.008841882	0.007873506
1997	60	54	0.012898377	0.011818702	6	5.44	0.014677997	0.012342398
1998	60	49	0.010228951	0.009183161	10	8.00	0.018233427	0.015486742
1999	50	42	0.013423875	0.013039100	20	17.44	0.012306245	0.011744996

**Table 3:** Different length of intervals for DJI and S&P 500 problems.

Year	DJI				S&P 500			
	*θ*	θ*	*l*	l*	*θ*	θ*	*l*	l*
2000	50	43	0.013228300	0.01108264	8	6.7	0.013240505	0.011592404
2001	50	46	0.014497805	0.013398244	6	5.3	0.012414271	0.012831417
2002	50	47	0.013735529	0.011088423	6	4.9	0.012357930	0.013486344
2003	30	24	0.008995559	0.008396447	4	3.2	0.008832271	0.009465817
2004	30	28	0.004236985	0.003389588	3	2.9	0.004085710	0.003368568
2005	30	26	0.004900489	0.004720391	3	2.4	0.004557089	0.003645671
2006	30	26	0.004925252	0.004640202	6	5.3	0.004986411	0.004389129
2007	40	37	0.007309639	0.007847711	8	7.4	0.007991680	0.006593344
2008	80	67	0.023692012	0.026353610	10	8.2	0.025768770	0.020615016
2009	40	32	0.014316920	0.015253536	6	4.9	0.015446702	0.018357362

**Table 4:** Different results on TAIEX, NASDAQ, DJI, and S&P 500 for original data (Yu [22]).

	Length	Adaptation	Year										Average
			1990	1991	1992	1993	1994	1995	1996	1997	1998	1999	
TAIEX	*θ*	Ne	273.9	67.1	55.0	105.1	134.4	82.9	54.5	148.8	152.6	122.9	119.7
	*θ*	I	241.2	57.4	49.9	104.1	109.0	76.6	53.8	141.4	133.7	118.0	108.5
	*θ*	II	242.0	59.1	50.1	104.4	110.4	75.8	53.6	141.7	132.3	118.9	108.8
	θ*	Ne	234.6	78.3	51.5	101.1	130.9	81.6	53.6	143.3	164.4	146.2	118.5
	θ*	I	221.0	66.5	49.5	100.8	112.6	76.7	53.2	137.9	142.5	125.8	108.6
	θ*	II	221.2	68.3	49.3	101.0	114.0	77.0	53.2	137.3	142.7	126.2	109.0

NASDAQ	*θ*	Ne	7.1	6.4	4.7	6.9	7.5	12.0	11.0	26.4	33.0	50.3	16.5
	*θ*	I	6.2	6.3	4.5	6.4	6.8	11.3	10.7	22.7	31.9	50.1	15.7
	*θ*	II	6.2	6.3	4.4	6.4	6.6	11.4	10.7	22.9	32.1	50.1	15.7
	θ*	Ne	6.6	7.0	5.1	6.7	7.3	11.2	10.7	28.1	32.0	49.9	16.5
	θ*	I	6.0	6.7	4.8	6.4	6.8	11.0	10.6	25.2	31.2	49.9	15.9
	θ*	II	6.0	6.6	4.8	6.7	6.9	10.9	10.4	25.2	31.9	49.9	15.9

	Length	Adaptation	Year										Average
			2000	2001	2002	2003	2004	2005	2006	2007	2008	2009	

DJI	*θ*	Ne	138.2	198.0	148.0	67.4	77.9	65.0	48.6	188.1	585.3	85.9	160.2
	*θ*	I	135.0	146.1	133.3	65.6	73.1	55.2	49.4	176.8	412.8	84.8	133.2
	*θ*	II	129.6	150.7	133.8	65.7	74.3	56.4	48.6	177.4	418.3	85.1	134.0
	θ*	Ne	138.6	203.0	156.1	66.0	73.4	61.3	54.6	182.1	564.1	83.4	158.3
	θ*	I	136.8	168.3	139.9	64.7	70.1	54.6	54.0	174.2	417.3	83.0	136.3
	θ*	II	135.9	171.7	139.0	64.9	70.0	54.6	53.7	174.9	419.7	83.1	136.8

S&P 500	*θ*	Ne	29.1	15.4	14.2	7.0	7.3	7.0	7.8	19.5	42.2	12.2	16.2
	*θ*	I	23.3	14.2	12.9	7.0	7.2	6.6	7.1	19.8	37.5	11.2	14.7
	*θ*	II	24.4	14.1	13.1	7.0	7.2	6.6	7.1	19.9	37.6	11.1	14.8
	θ*	Ne	28.4	14.9	18.0	7.8	7.6	6.4	7.2	21.2	43.1	11.6	16.6
	θ*	I	24.6	14.3	16.9	7.6	7.6	6.3	6.9	21.0	37.1	11.1	15.3
	θ*	II	25.3	14.2	16.6	7.8	7.6	6.3	6.9	21.0	37.8	11.1	15.4

**Table 5:** Different results on TAIEX, NASDAQ, DJI, and S&P 500 for preprocessed data (Yu [22]).

	Length	Adaptation	Year										Average
			1990	1991	1992	1993	1994	1995	1996	1997	1998	1999	
TAIEX	*l*	Ne	175.7	45.3	41.9	106.8	93.4	64.1	50.5	137.4	115.8	103.3	93.4
	*l*	I	171.8	43.6	41.4	104.4	93.9	64.8	51.6	136.9	114.9	105.8	92.9
	*l*	II	170.1	43.3	41.6	104.6	95.2	65.1	51.6	137.4	116.4	105.7	93.1
	l*	Ne	176.9	43.9	44.7	102.2	93.0	62.2	48.6	146.8	116.8	98.9	93.4
	l*	I	170.7	42.9	43.1	101.6	92.9	62.5	49.2	144.2	113.7	100.8	92.2
	l*	II	171.1	43.1	43.1	101.6	95.3	62.3	48.8	144.9	114.4	100.8	92.5

NASDAQ	*l*	Ne	4.2	6.8	4.1	5.2	5.7	12.1	9.9	19.8	34.1	46.9	14.9
	*l*	I	4.8	6.7	4.0	5.2	5.6	11.7	10.2	20.2	32.4	49.0	15.0
	*l*	II	4.8	6.4	4.1	5.3	5.6	12.0	10.2	20.2	34.4	47.2	15.0
	l*	Ne	5.3	6.7	4.0	5.2	5.4	11.5	9.8	19.8	30.6	48.4	14.7
	l*	I	5.2	6.8	4.0	5.2	5.4	11.4	10.0	20.5	30.4	50.1	14.9
	l*	II	5.2	6.5	4.0	5.2	5.4	11.5	10.0	20.6	30.2	48.4	14.7

	Length	Adaptation	Year										Average
			2000	2001	2002	2003	2004	2005	2006	2007	2008	2009	

DJI	*l*	Ne	144.5	100.2	108.6	60.7	66.0	49.1	56.2	160.8	306.1	79.3	113.2
	*l*	I	142.8	98.0	108.4	60.7	65.3	47.4	55.2	162.4	290.5	85.3	111.6
	*l*	II	142.3	96.9	109.9	62.1	65.5	47.5	55.3	162.9	299.0	86.1	112.7
	l*	Ne	136.8	95.3	110.7	58.5	64.0	52.0	55.8	161.6	296.3	81.8	111.3
	l*	I	136.1	94.5	109.9	58.4	63.3	49.8	55.1	162.3	296.0	84.8	111.0
	l*	II	136.3	94.8	111.7	59.0	64.1	49.8	55.3	163.1	301.7	84.8	112.0

S&P 500	*l*	Ne	19.3	11.5	11.8	6.9	7.4	6.4	7.0	20.5	36.3	8.9	13.6
	*l*	I	19.7	11.3	11.8	6.9	7.3	6.3	6.8	20.6	35.1	9.9	13.6
	*l*	II	19.6	11.3	11.8	6.9	7.3	6.3	7.1	20.7	35.0	9.9	13.6
	l*	Ne	19.7	11.6	11.7	6.6	7.7	6.1	6.9	20.3	31.8	9.0	13.1
	l*	I	20.3	11.4	11.6	6.6	7.5	6.0	6.7	20.4	31.9	9.5	13.2
	l*	II	20.1	11.4	11.9	6.7	7.7	6.1	6.7	20.6	31.6	9.5	13.2
